# High-Accuracy Parallel Neural Networks with Hard Constraints for a Mixed Stokes/Darcy Model

**DOI:** 10.3390/e27030275

**Published:** 2025-03-06

**Authors:** Zhulian Lu, Junyang Zhang, Xiaohong Zhu

**Affiliations:** Department of Mathematics, Jinan University, Guangzhou 510632, China

**Keywords:** mixed Stokes/Darcy model, physics-informed neural networks, hard constraints, soft constraints

## Abstract

In this paper, we study numerical algorithms based on Physics-Informed Neural Networks (PINNs) for solving a mixed Stokes/Darcy model that describes a fluid flow coupled with a porous media flow. A Hard Constrained Parallel PINN (HC-PPINN) is proposed for the mixed model, in which the boundary conditions are enforced by modified the neural network architecture. Numerical experiments with different settings are conducted to demonstrate the accuracy and efficiency of our method by comparing it with the methods based on vanilla PINNs for the mixed model.

## 1. Introduction

In the real world, there are many applications involving the interaction of different physical processes in different subdomains of the problem domain. We focus on the mixed Stokes/Darcy model, which models the motion of fluid between the surface and subsurface regions. The behavior of the fluid is characterized by different partial differential equations, with the Stokes equations describing the behavior in the surface region and Darcy’s law governing the behavior in the subsurface region [1,2,3,4]. The two regions are coupled by the appropriate interface conditions that ensure the conservation of mass and the balance of normal forces across the interface, and the Beavers–Joseph–Saffman interface condition, which states that the shear stress along the interface is proportional to the slip velocity along the interface [5,6,7,8]. The mixed Stokes/Darcy model boasts an extensive array of applications, e.g., groundwater systems [9,10], industrial filtration [11,12,13], blood flow in tumors [14,15], etc.

The traditional numerical techniques for resolving the mixed Stokes/Darcy model are extensively documented in the literature [2,3,16,17,18,19,20,21]. In general, there are two main approaches: one approach involves solving the coupled problem directly [22,23], while the other involves decoupling the mixed model first and then applying appropriate local solvers independently [24,25,26]. These methods can be difficult to solve in the face of irregular regions and curved interfaces.

Over the last two decades, deep learning has achieved extraordinary success in a range of domains, such as computer vision and natural language processing [27]. Solving Partial Differential Equations (PDEs) via deep learning has recently surfaced as a promising topic known as Scientific Machine Learning (SciML) [28]. A representative class of results has been presented, including but not limited to the following: In 2018, M. Raissi et al. devised a deep-learning framework, namely, a Physics-Informed Neural Network (PINN), to solve the forward and inverse problems of partial differential equations, and utilized it to study the equations of hydrodynamics and their inverse processes [29,30]. J. Sirignano et al. proposed the use of deep neural networks to solve high-dimensional partial differential equations, called the Deep Galerkin Method (DGM), and gave a theoretical analysis of the approximation performance of neural networks [31]. In [32], the Deep Ritz Method has been proposed by authors to deal with variational problems. In 2020, Y. Zang et al. proposed solving high-dimensional partial differential equations over irregular domains using weak adversarial networks [33]. In 2021, S. Dong et al. solved linear and nonlinear partial differential equations using domain decomposition and local extreme learning machines [34], etc. These neural network-based PDE solving methods are widely popular due to their universal approximation properties [35,36]. Compared with traditional grid-based methods, deep learning to solve PDE is a grid-free method that utilizes automatic differentiation [29], which can break the curse of dimensionality [37,38].

Among these methods, PINNs is one of the most popular methods. There are currently many variants of PINNs, such as variational hp-VPINNs [39], conservative PINNs (cPINNs) [40], extended PINNs (XPINNs) [41], Parallel PINNs (PPINNs), etc. However, the constraints for boundary and initial conditions in most of the PINN-based methods are soft constraints. In order to strictly enforce the boundary and initial conditions, Refs.  [42,43] devised a PINN-based architecture that could enhance both the precision and generalization ability of the neural network.

According to the literature research, most of the current studies on deep learning for solving PDEs are for problems controlled by a single set of physics equations, while there are fewer studies on the Stokes/Darcy coupled problem [44]. In 2022, R. Pu et al. investigated the steady Stokes/Darcy coupled problem by using PINNs and proposed a strategy to improve the accuracy [45]. In 2023, J. Yue et al. proposed Coupled Deep Neural Networks (CDNNs) for solving the time-dependent Stokes/Darcy coupling problem [46]. Ref. [47] investigated the neural network solution method for the forward and backward problems of the Navier–Stokes/Darcy coupling problem based on PINNs. However, the fitting accuracies of the existing studies are low, with the relative $L_{2}$ error remaining between 10^−2^ and 10^−4^, and these studies primarily focus on regular regions and straight-line interfaces.

In this paper, to improve neural network accuracy in solving Stokes/Darcy coupled problems, we first design a parallel physics-informed neural network, namely, Parallel PINNs (PPINNs). Then, we modify the network architecture to enforce boundary conditions, and at the same time incorporate the control equations as well as the interface equations into the loss with soft constraints for training; we call it HC-PPINNs. Specifically, the training of HC-PPINNs only needs to be driven by minimizing the loss of the governing equations and interface equations and does not need to be data driven. Since it is not to be data driven, the training cost is greatly reduced. In addition, HC-PPINNs achieve higher accuracy compared to the methods based on vanilla PINNs, including the Parallel PINNs (PPINNs) and CDNNs in [46]. Furthermore, HC-PPINNs can also keep good performance in both irregular regions and curved interfaces. The performance and accuracy of our method, HC-PPINNs, are demonstrated by five examples.

The structure of the paper is as follows. The coupling problem is presented in Section 2. In Section 3, we present the architecture of our PPINN and HC-PPINN. Section 4 demonstrates the performance of HC-PPINNs by five examples. The last section concludes the paper.

## 2. Problem Formulation

**Figure 1:**
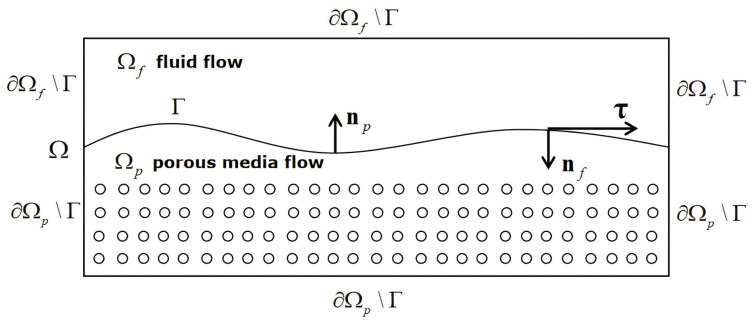
The global domain Ω consisting of the fluid flow region $\Omega_{f}$ and the porous media flow region $\Omega_{p}$, separated by the interface Γ.

We consider a coupled fluid flow and porous media flow in a bounded domain $\Omega \subset R^{d}(d=2$ or 3), which consists of a fluid flow in $\Omega_{f}$ and a porous media flow in $\Omega_{p}$, separated by an interface Γ (see Figure 1), where $\Omega_{f}\cup \Omega_{p}=\Omega$, $\Omega_{f}\cap \Omega_{p}=\emptyset$, and ${\bar{\Omega }}_{f}\cap {\bar{\Omega }}_{p}=\Gamma$. Let $n_{f}$ and $n_{p}$ be the unit outward normal vectors on the boundaries of $\Omega_{f}$ and $\Omega_{p}$, respectively, and $\tau_{i}$, i=1,⋯,d−1, the unit tangential vectors on the interface Γ. Then, we have $n_{p}=-n_{f}$ on Γ.

The Stokes equations are used to describe the motion of the fluid flow in $\Omega_{f}$

(1) $$\begin{array}{ccc} -\nu \Delta u_{f}+\nabla p_{f}=g_{f} & & \mathrm{in}\Omega_{f}, \end{array}$$

(2) $$\begin{array}{ccc} \nabla \;\cdot \;u_{f}=0 & & \mathrm{in}\Omega_{f}, \end{array}$$

where $u_{f}\left(x\right)$ is the velocity of the fluid flow in $\Omega_{f}$, $p_{f}\left(x\right)$ is the pressure, and $g_{f}$ is the external force.

The following equations are used to describe the motion of the porous media flow in $\Omega_{p}$:

(3) $$\begin{array}{ccc} \nabla \;\cdot \;q=g_{p} & & \mathrm{in}\Omega_{p}, \end{array}$$

(4) $$\begin{array}{ccc} q=-K\nabla \varphi & & \mathrm{in}\Omega_{p}\;\;\left({\mathrm{Darcy}}^{\prime }s\mathrm{law}\right), \end{array}$$

(5) $$\begin{array}{ccc} u_{p}=\frac{q}{n} & & \mathrm{in}\Omega_{p}, \end{array}$$

where q is the specific discharge, which is defined as the volume of the fluid flowing per unit time through a unit cross-sectional area normal to the direction of the flow. $\varphi \left(x\right)=z+\frac{p_{p}}{\rho g}$ is the piezometric head, which is the sum of elevation head *z* and the pressure head. $p_{p}$ is the pressure of the fluid in $\Omega_{p}$, ρ is the density of the fluid, and *g* is the gravitational acceleration. $u_{p}$ is the fluid velocity in $\Omega_{p}$, K is the hydraulic conductivity tensor, *n* is the volumetric porosity, and $g_{p}$ is the source term. For simplicity, we assume z=0 and the porous media is homogeneous, i.e., K=diag(K,⋯,K) with $K\in L^{\infty }\left(\Omega_{p}\right)$, K>0. Then, the continuity Equation (3) in $\Omega_{p}$ can be written in the following form by using Darcy’s law (4):

(6) $$\begin{array}{ccc} -\nabla \;\cdot \;\left(K\nabla \varphi \right)=g_{p} & & \mathrm{in}\Omega_{p}. \end{array}$$

The interface coupling conditions are the important part in a mixed model. For the mixed Stokes/Darcy model, the following interface conditions are used in the literature [5,6,7,8]:

(7) $$\begin{array}{ccc} u_{f}\;\cdot \;n_{f}+u_{p}\;\cdot \;n_{p}=0 & & \mathrm{on}\Gamma , \end{array}$$

(8) $$\begin{array}{ccc} p_{f}-\nu n_{f}\frac{\partial u_{f}}{\partial n_{f}}=\rho g\varphi & & \mathrm{on}\Gamma , \end{array}$$

(9) $$\begin{array}{ccc} -\nu \tau_{i}\frac{\partial u_{f}}{\partial n_{f}}=\frac{\alpha }{\sqrt{\tau_{i}\;\cdot \;K\tau_{i}}}u_{f}\;\cdot \;\tau_{i},\\ \;i=1,\cdots ,d-1 & & \mathrm{on}\Gamma , \end{array}$$

where α>0 is a parameter depending on the properties of the porous medium and should be experimentally determined. The first interface condition (7) is the mass conservation across the interface Γ. Using (4) and (5), it can be rewritten as

(10) $$\begin{array}{ccc} u_{f}\;\cdot \;n_{f}=\frac{K}{n}\frac{\partial \varphi }{\partial n_{p}} & & \mathrm{on}\Gamma . \end{array}$$

The second interface condition (8) is the balance of the normal forces across the interface. The third one (9), known as the Beavers–Joseph–Saffman law [5,7,17], states that the slip velocity along the interface is proportional to the shear stress along the interface.

For the convenience of the following discussion, the Dirichlet Boundary Conditions (BCs) are considered for the mixed model:

(11) $$\begin{array}{ccc} u_{f}=u_{f}^{b} & & \mathrm{on}\partial \Omega_{f}\setminus \Gamma , \end{array}$$

(12) $$\begin{array}{ccc} \varphi =\varphi^{b} & & \mathrm{on}\partial \Omega_{p}\setminus \Gamma . \end{array}$$

Besides the Dirichlet BCs, the mixed Dirichlet and Neumann BCs are set up in the later numerical examples.

In summary, the mixed Stokes/Darcy model consists of the control equations, including the Stokes Equations (1) and (2) Darcy’s law (6), and the interface conditions (8)–(10) and the boundary conditions (11) and (12).

## 3. Methodology

In this section, the mixed Stokes/Darcy model is first solved using a parallel physics-informed neural networks that is mainly based on the vanilla PINNs [29]. Then, a high-accuracy parallel neural network with hard constraints for the boundary conditions is presented for the mixed model.

### 3.1. Parallel Physics-Informed Neural Networks

**Figure 2:**
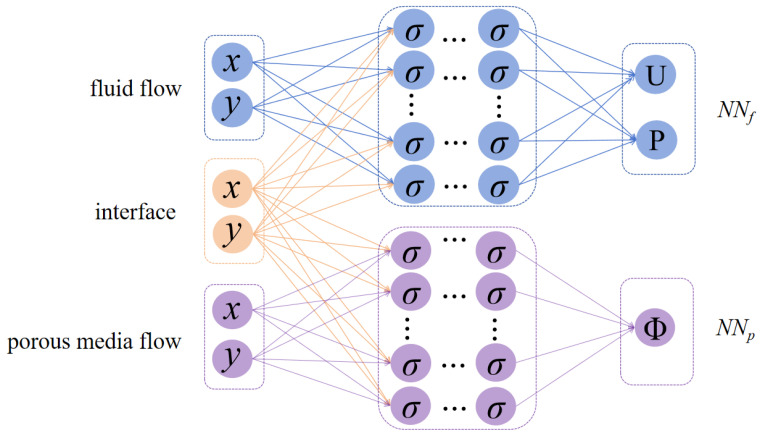
Diagram of the PPINNs architecture.

In [41], the authors divided the solution region into many sub-regions and utilized separate networks inside each sub-region to solve nonlinear PDEs on domains with arbitrary complex geometries. Inspired by this approach, we present a parallel Physics-Informed Neural Network, called PPINN, for the mixed Stokes/Darcy model, in which there is one neural network for the fluid flow region $\Omega_{f}$, and another for the porous media flow region $\Omega_{p}$. Figure 2 displays the diagram of the PPINN architecture.

Let ${\left\{x_{f}^{\left(i\right)}\right\}}_{i=1}^{N_{f}}$ in $\Omega_{f}$, ${\left\{x_{p}^{\left(i\right)}\right\}}_{i=1}^{N_{p}}$ in $\Omega_{p}$, ${\left\{x_{\Gamma }^{\left(i\right)}\right\}}_{i=1}^{N_{\Gamma }}$ on Γ, and boundary points ${\left\{x_{bf}^{\left(i\right)}\right\}}_{i=1}^{N_{bf}}$ on $\partial \Omega_{f}\setminus \Gamma$, ${\left\{x_{bp}^{\left(i\right)}\right\}}_{i=1}^{N_{bp}}$ on $\partial \Omega_{p}\setminus \Gamma$ be the set of randomly selected collocation points. Here, $N_{f}$, $N_{p}$, and $N_{\Gamma }$ are the numbers of collocation points in the interior of the domain $\Omega_{f}$, $\Omega_{p}$, and Γ, respectively; $N_{bf}$ and $N_{bp}$ are the numbers of the collocation points on the boundary of $\Omega_{f}$ and $\Omega_{p}$, respectively.

Let $U(x;\theta_{f})$, $P(x;\theta_{f})$, and $\Phi (x;\theta_{p})$ be the neural network approximation solutions of $u_{f}$, $p_{f}$, and φ, respectively, where $\theta_{f}$ denotes the parameters of the neural network $NN_{f}$ for the fluid flow and $\theta_{p}$ the parameters of the neural network $NN_{p}$ for the porous media flow. Based on the vanilla PINNs, we restrict these two neural networks to satisfy the Stokes/Dacy problem by using a PDE-informed loss function, where the boundary conditions are treated in a “soft” manner, namely soft constraints, through a loss function.

Then, the problem of PPINNs for the mixed Stokes/Darcy model can be described by the following minimization problem of the loss function $L(\theta_{f},\theta_{p})$ with respect to the parameters $\theta_{f}$ and $\theta_{p}$,

(13) $$\begin{array}{c} \min_{\theta_{f},\theta_{p}}L\left(\theta_{f},\theta_{p}\right)=\lambda_{f}L_{\Omega_{f}}\left(\theta_{f}\right)+\lambda_{p}L_{\Omega_{p}}\left(\theta_{p}\right)+\lambda_{\Gamma }L_{\Gamma }\left(\theta_{f},\theta_{p}\right), \end{array}$$

where

$$\begin{array}{cc} \; & L_{\Omega_{f}}\left(\theta_{f}\right)=\underset{L_{\Omega_{f},pde}}{\underset{︸}{\frac{1}{N_{f}}\sum_{i=1}^{N_{f}}\left({\left|G_{\Omega_{f},1}\left[U,P\right]\left(x_{f}^{\left(i\right)};\theta_{f}\right)\right|}^{2}+{\left|G_{\Omega_{f},2}\left[U\right]\left(x_{f}^{\left(i\right)};\theta_{f}\right)\right|}^{2}\right)}} \end{array}$$

(14) $$\begin{array}{cc} \; & \;+\underset{L_{\Omega_{f},bc}}{\underset{︸}{\frac{1}{N_{bf}}\sum_{i=1}^{N_{bf}}{\left|U\left(x_{bf}^{\left(i\right)};\theta_{f}\right)-u_{f}^{b}\left(x_{bf}^{\left(i\right)}\right)\right|}^{2}}}+\underset{L_{\Omega_{f},pressure}}{\underset{︸}{\frac{1}{N_{bf}}\sum_{i=1}^{N_{bf}}{\left|P\left(x_{bf}^{\left(i\right)};\theta_{f}\right)-p_{f}^{\left(i\right)}\right|}^{2}}}, \end{array}$$

(15) $$\begin{array}{cc} \; & L_{\Omega_{p}}\left(\theta_{p}\right)=\underset{L_{\Omega_{p},pde}}{\underset{︸}{\frac{1}{N_{p}}\sum_{i=1}^{N_{p}}{\left|G_{\Omega p}\left[\Phi \right]\left(x_{p}^{\left(i\right)};\theta_{p}\right)\right|}^{2}}}+\underset{L_{\Omega_{p},bc}}{\underset{︸}{\frac{1}{N_{bp}}\sum_{i=1}^{N_{bp}}{\left|\Phi \left(x_{bp}^{\left(i\right)};\theta_{p}\right)-\varphi^{b}\left(x_{bp}^{\left(i\right)}\right)\right|}^{2}}},\\ \; & L_{\Gamma }\left(\theta_{f},\theta_{p}\right)=\frac{1}{N_{\Gamma }}\sum_{i=1}^{N_{\Gamma }}\left({\left|G_{\Gamma ,1}\left[U,\Phi \right]\left(x_{\Gamma }^{\left(i\right)};\theta_{f},\theta_{p}\right)\right|}^{2}+{\left|G_{\Gamma ,2}\left[U,P,\Phi \right]\left(x_{\Gamma }^{\left(i\right)};\theta_{f},\theta_{p}\right)\right|}^{2}\right. \end{array}$$

(16) $$\begin{array}{c} \left.+{\left|G_{\Gamma ,3}\left[U\right]\left(x_{\Gamma }^{\left(i\right)};\theta_{f}\right)\right|}^{2}\right) \end{array}$$

with

(17) $$\begin{array}{c} \left\{\begin{array}{c} G_{\Omega_{f},1}\left[U,P\right]=-\nu \Delta U+\nabla P-g_{f},\\ G_{\Omega_{f},2}\left[U\right]=\nabla \;\cdot \;U,\\ G_{\Omega p}\left[\Phi \right]=-\nabla \;\cdot \;\left(K\nabla \Phi \right)-g_{p},\\ G_{\Gamma ,1}\left[U,\Phi \right]=U\;\cdot \;n_{f}-\frac{K}{n}\frac{\partial \Phi }{\partial n_{p}},\\ G_{\Gamma ,2}\left[U,P,\Phi \right]=P-\nu n_{f}\frac{\partial U}{\partial n_{f}}-\rho g\Phi ,\\ G_{\Gamma ,3}\left[U\right]=\sum_{i=1}^{d-1}\left(\nu \tau_{i}\frac{\partial U}{\partial n_{f}}+\frac{\alpha }{\sqrt{\tau_{i}\;\cdot \;K\tau_{i}}}U\;\cdot \;\tau_{i}\right),\;i=1,\cdots ,d-1. \end{array}\right. \end{array}$$

The total loss $L(\theta_{f},\theta_{p})$ includes three loss functions, $L_{\Omega_{f}}\left(\theta_{f}\right)$, $L_{\Omega_{p}}\left(\theta_{p}\right)$, and $L_{\Gamma }\left(\theta_{f},\theta_{p}\right)$, from the fluid flow region $\Omega_{f}$, the porous media region $\Omega_{p}$, and the interface Γ, respectively. The positive parameters $\lambda_{f}$, $\lambda_{p}$, and $\lambda_{\Gamma }$ represent the weights of the loss functions in $\Omega_{f}$, $\Omega_{p}$, and Γ, respectively. These weights ensure that the different components of the loss function are balanced, which can improve the convergence of the PINN-based methods [48,49]. The terms $L_{\;\cdot \;,pde}\left(\;\right)$ are the PDE-informed loss functions from the fluid flow region $\Omega_{f}$ and the porous media region $\Omega_{p}$. The terms $L_{\;\cdot \;,bc}\left(\;\right)$ are the loss from the boundary conditions. Each of the loss $L_{\Omega_{f}}\left(\theta_{f}\right)$ and $L_{\Omega_{p}}\left(\theta_{f}\right)$ includes the PDE-informed loss and the loss from the boundary conditions. For the Stokes equation, the pressure field can only be determined up to a constant, and additional constraints need to be introduced. These constraints typically include fixing the pressure at a specific reference point or incorporating a regularization term to ensure that the mean pressure over the domain is zero [45]. To improve the pressure approximation, we take the approach presented in reference [50] by adding a pressure training loss function $L_{\Omega_{f},pressure}$ in the loss $L_{\Omega_{f}}\left(\theta_{f}\right)$. The pressure data on the boundary $p_{f}^{\left(i\right)}$ should be given by the additional information about the pressure. We denote the pressure data by $p_{f}^{b}={\left\{p_{f}^{\left(i\right)}\right\}}_{i=1}^{N_{bf}}$. Note that, for the case of in the absence of available pressure data, the pressure-related loss function is omitted from the formulation, and the constraints mentioned above could be imposed. In our numerical experiments, if there exist exact solutions for the coupled model, then the pressure data are given by the exact solution, such as in Examples 1, 2, 3, and 5. There is no exact solution for Example 4, where the pressure loss is omitted.

The main steps of PPINNs are presented as Algorithm 1.

**Algorithm 1** PPINNs for the mixed Stokes/Darcy model
**Input:** Randomly selected collocation points ${\left\{x_{f}^{\left(i\right)}\right\}}_{i=1}^{N_{f}}$, ${\left\{x_{p}^{\left(i\right)}\right\}}_{i=1}^{N_{p}}$, ${\left\{x_{\Gamma }^{\left(i\right)}\right\}}_{i=1}^{N_{\Gamma }}$, ${\left\{x_{bf}^{\left(i\right)}\right\}}_{i=1}^{N_{bf}}$, and ${\left\{x_{bp}^{\left(i\right)}\right\}}_{i=1}^{N_{bp}}$; The pressure data $p_{f}^{b}={\left\{p_{f}^{\left(i\right)}\right\}}_{i=1}^{N_{bf}}$ if available; The maximum number of iterations *M*; The learning rate $\alpha_{f}$ and $\alpha_{p}$.**Output:** $\theta_{f}^{n+1}$ and $\theta_{p}^{n+1}$.
  1:n = 1.  2:Initialize the neural network parameters $\theta_{f}^{n}$ and $\theta_{p}^{n}$.  3:**while** n<=M **do**      read current;      compute $L\left(\theta_{f}^{n},\theta_{p}^{n}\right)=\lambda_{f}L_{\Omega_{f}}\left(\theta_{f}^{n}\right)+\lambda_{p}L_{\Omega_{p}}\left(\theta_{p}^{n}\right)+\lambda_{\Gamma }L_{\Gamma }\left(\theta_{f}^{n},\theta_{p}^{n}\right)$;      update            $\theta_{f}^{n+1}=\theta_{f}^{n}-\alpha_{f}\nabla_{\theta_{f}^{n}}L\left(\theta_{f}^{n},\theta_{p}^{n}\right)$;            $\theta_{p}^{n+1}=\theta_{p}^{n}-\alpha_{p}\nabla_{\theta_{p}^{n}}L\left(\theta_{f}^{n},\theta_{p}^{n}\right)$;      n=n+1;  4:
**end while**



### 3.2. Hard Constrained PPINNs

In PPINNs, the loss function $L(\theta_{f},\theta_{p})$ will continue to decrease with optimization and gradually approach zero. For the known Dirichlet BCs $u_{f}^{b}$ and $\varphi^{b}$, and the data $p_{f}^{b}$ if available, the loss $L_{\Omega_{f},bc}$, $L_{\Omega_{p},bc}$ and $L_{\Omega_{f},pressure}$ should be able to reach zero theoretically during optimization. However, this may not be achieved in the numerical optimization of PPINNs, which reduces the accuracy of the PPINNs. Therefore, in order to make full use of the known data, we designed a new network architecture based on PPINNs to enforce Dirichlet BCs $u_{f}^{b}$ and $\varphi^{b}$, as well as the data $p_{f}^{b}$, ensuring that the loss of the new network from boundary remains 0. In this way, the boundary conditions and the pressure data are treated in a ”hard” manner, called hard constraints [42,43].

**Figure 3:**
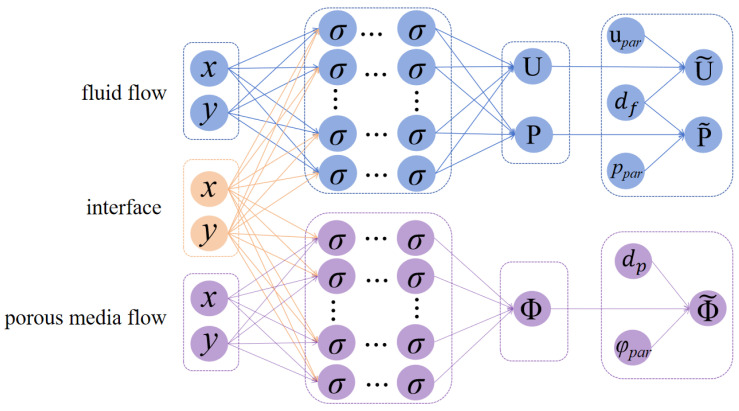
Diagram of the HC-PPINNs architecture.

We strictly impose the Dirichlet BCs by modifying the neural network architecture. Specifically, we construct the neural network solutions as

(18) $$\begin{array}{cc} & \tilde{U}\left(x;{\tilde{\theta }}_{f}\right)=u_{par}\left(x\right)+d_{f}\left(x\right)U\left(x;{\tilde{\theta }}_{f}\right), \end{array}$$

(19) $$\begin{array}{cc} & \tilde{P}\left(x;{\tilde{\theta }}_{f}\right)=p_{par}\left(x\right)+d_{f}\left(x\right)P\left(x;{\tilde{\theta }}_{f}\right), \end{array}$$

(20) $$\begin{array}{cc} & \tilde{\Phi }\left(x;{\tilde{\theta }}_{p}\right)=\varphi_{par}\left(x\right)+d_{p}\left(x\right)\Phi \left(x;{\tilde{\theta }}_{p}\right), \end{array}$$

where $\tilde{U}\left(x;{\tilde{\theta }}_{f}\right)$, $\tilde{P}\left(x;{\tilde{\theta }}_{f}\right)$, $\tilde{\Phi }\left(x;{\tilde{\theta }}_{p}\right)$ are the final outputs of the networks; see Figure 3. Here, $u_{par}\left(x\right)$ and $\varphi_{par}\left(x\right)$ are solutions that just satisfy the Dirichlet BCs, respectively: $u_{par}{|}_{\partial \Omega_{f}\setminus \Gamma }=u_{f}^{b}$ and $\varphi_{par}{|}_{\partial \Omega_{p}\setminus \Gamma }=\varphi^{b}$. While $p_{par}\left(x\right)$ satisfies the pressure data: $p_{par}{|}_{\partial \Omega_{f}\setminus \Gamma }=p_{f}^{b}$ if the pressure data is available. Analogous to the pressure treatment methodology employed in Algorithm 1 for the mixed Stokes/Darcy model, the hard constraint on pressure (19) will be conducted if the pressure data are available, otherwise, this constraint will be omitted. Specifically, in the later numerical experiments, the hard constraint on pressure will only be imposed in Examples 1, 2, 3, and 5. $d_{i}\left(x\right)\left(i=f,p\right)$ is a smooth distance function satisfying the following two conditions:

$$\left\{\begin{array}{cc} d_{i}\left(x\right)=0, & x\in \partial \Omega_{i}\setminus \Gamma ,\\ d_{i}\left(x\right)>0, & x\in \Omega_{i}, \end{array}\right.$$

where $d_{i}\left(x\right)\left(i=f,p\right)$ needs to be constructed case-by-case. For example, when $\Omega_{f}=\left(0,1\right)\times \left(1,2\right)$, $\Omega_{p}=\left(0,1\right)\times \left(0,1\right)$ and $\Gamma =\left(0,1\right)\times \left\{1\right\}$, we can choose $d_{f}\left(x\right)=x\left(x-1\right)\left(y-2\right)$ and $d_{p}\left(x\right)=x\left(x-1\right)y$, where x=(x,y). This example will be used in Section 4. For complex regions, please refer to [51] for the construction method of $d_{i}\left(x\right)$.

Then, the loss function here is transformed into a form without simulation data:

(21) $$\min_{{\tilde{\theta }}_{f},{\tilde{\theta }}_{p}}J\left({\tilde{\theta }}_{f},{\tilde{\theta }}_{p}\right)={\tilde{\lambda }}_{f}J_{\Omega_{f}}\left({\tilde{\theta }}_{f}\right)+{\tilde{\lambda }}_{p}J_{\Omega_{p}}\left({\tilde{\theta }}_{p}\right)+{\tilde{\lambda }}_{\Gamma }J_{\Gamma }\left({\tilde{\theta }}_{f},{\tilde{\theta }}_{p}\right),$$

where

(22) $$\begin{array}{cc} \; & J_{\Omega_{f}}\left({\tilde{\theta }}_{f}\right)=\frac{1}{N_{f}}\sum_{i=1}^{N_{f}}\left({\left|G_{\Omega_{f},1}\left[\tilde{U},\tilde{P}\right]\left(x_{f}^{\left(i\right)};{\tilde{\theta }}_{f}\right)\right|}^{2}+{\left|G_{\Omega_{f},2}\left[\tilde{U},\tilde{P}\right]\left(x_{f}^{\left(i\right)};{\tilde{\theta }}_{f}\right)\right|}^{2}\right), \end{array}$$

(23) $$\begin{array}{cc} \; & J_{\Omega_{p}}\left({\tilde{\theta }}_{p}\right)=\frac{1}{N_{p}}\sum_{i=1}^{N_{p}}{\left|G_{\Omega_{p}}\left[\tilde{\Phi }\right]\left(x_{p}^{\left(i\right)};{\tilde{\theta }}_{p}\right)\right|}^{2},\\ \; & J_{\Gamma }\left({\tilde{\theta }}_{f},{\tilde{\theta }}_{p}\right)=\frac{1}{N_{\Gamma }}\sum_{i=1}^{N_{\Gamma }}\left({\left|G_{\Gamma ,1}\left[\tilde{U},\tilde{\Phi }\right]\left(x_{\Gamma }^{\left(i\right)};{\tilde{\theta }}_{f},{\tilde{\theta }}_{p}\right)\right|}^{2}+{\left|G_{\Gamma ,2}\left[\tilde{U},\tilde{P},\tilde{\Phi }\right]\left(x_{\Gamma }^{\left(i\right)};{\tilde{\theta }}_{f},{\tilde{\theta }}_{p}\right)\right|}^{2}\right. \end{array}$$

(24) $$\begin{array}{c} \left.+{\left|G_{\Gamma ,3}\left[\tilde{U}\right]\left(x_{\Gamma }^{\left(i\right)};{\tilde{\theta }}_{f}\right)\right|}^{2}\right), \end{array}$$

where the definitions of the operators $G_{\Omega_{f},1}\left[\;\cdot \;,\;\cdot \;\right]$, $G_{\Omega_{f},2}\left[\;\cdot \;,\;\cdot \;\right]$, $G_{\Omega_{p}}\left[\;\cdot \;\right]$, $G_{\Gamma ,1}\left[\;\cdot \;,\;\cdot \;\right]$, $G_{\Gamma ,2}\left[\;\cdot \;,\;\cdot \;,\;\cdot \;\right]$, and $G_{\Gamma ,3}\left[\;\cdot \;\right]$ are in Equation (17). The positive parameters ${\tilde{\lambda }}_{f}$, ${\tilde{\lambda }}_{p}$, and ${\tilde{\lambda }}_{\Gamma }$ represent the weights of the loss functions in $\Omega_{f}$, $\Omega_{p}$, and Γ, respectively.

We call the above parallel PINNs with hard constraints HC-PPINNs, and the main steps are listed in Algorithm 2.

**Algorithm 2** HC-PPINNs for the mixed Stokes/Darcy model
**Input:** Randomly selected collocation points ${\left\{x_{f}^{\left(i\right)}\right\}}_{i=1}^{N_{f}}$, ${\left\{x_{p}^{\left(i\right)}\right\}}_{i=1}^{N_{p}}$, ${\left\{x_{\Gamma }^{\left(i\right)}\right\}}_{i=1}^{N_{\Gamma }}$, The maximum number of iterations $\tilde{M}$; The learning rate ${\tilde{\alpha }}_{f}$ and ${\tilde{\alpha }}_{p}$.**Output:** ${\tilde{\theta }}_{f}^{n+1}$ and ${\tilde{\theta }}_{p}^{n+1}$.
  1:n = 1.  2:Initialize the neural network parameters ${\tilde{\theta }}_{f}^{n}$ and ${\tilde{\theta }}_{p}^{n}$.  3:**while** $n<=\tilde{M}$ **do**      read current;      compute $J\left({\tilde{\theta }}_{f}^{n},{\tilde{\theta }}_{p}^{n}\right)={\tilde{\lambda }}_{f}J_{\Omega_{f}}\left({\tilde{\theta }}_{f}^{n}\right)+{\tilde{\lambda }}_{p}J_{\Omega_{p}}\left({\tilde{\theta }}_{p}^{n}\right)+{\tilde{\lambda }}_{\Gamma }J_{\Gamma }\left({\tilde{\theta }}_{f}^{n},{\tilde{\theta }}_{p}^{n}\right)$;      update            ${\tilde{\theta }}_{f}^{n+1}={\tilde{\theta }}_{f}^{n}-{\tilde{\alpha }}_{f}\nabla_{{\tilde{\theta }}_{f}^{n}}J\left({\tilde{\theta }}_{f}^{n},{\tilde{\theta }}_{p}^{n}\right)$;            ${\tilde{\theta }}_{p}^{n+1}={\tilde{\theta }}_{p}^{n}-{\tilde{\alpha }}_{p}\nabla_{{\tilde{\theta }}_{p}^{n}}J\left({\tilde{\theta }}_{f}^{n},{\tilde{\theta }}_{p}^{n}\right)$;      n=n+1;  4:
**end while**



## 4. Computational Results and Discussion

For illustrating the performance of the two neural network PPINNs and HC-PPINNs presented above, we present numerical results for five different settings of the mixed Stokes/Darcy model.

Our experiments are based on Python 3.8.19, TensorFlow 2.0.0, and Keras 2.3.1, and the computer is configured as an Intel(R) Core (TM) i5-8300H. If not specified, in the following numerical experiments the network $NN_{f}$ and $NN_{p}$ will use the same number of hidden layers and the same number of neurons in each hidden layer. We employ Xavier initialization, tanh activation function, a learning rate of 1 × 10^−4^, and iterations $M=\tilde{M}=$ 1 × 10^4^. For the collocation points, we take $N_{f}=N_{p}=400$, $N_{\Gamma }=100$, $N_{bf}=N_{bp}=300$. The weight parameters $\lambda_{i}$ and ${\tilde{\lambda }}_{i}$ (i=f,p,Γ) are all set to 1.

The following relative $L_{2}$ error between the neural network approximation solution *U* and the exact solution *u* will be used in the examples.

(25) $$\begin{array}{c} E\left(U,u\right)=\frac{\sum_{i=1}^{N}{\left|U^{\left(i\right)}-u^{\left(i\right)}\right|}^{2}}{\sum_{i=1}^{N}{\left|u^{\left(i\right)}\right|}^{2}}, \end{array}$$

where *N* represents the number of points in the test set. We employ a test set of 10,000 points with equi-spaced uniform distribution in $\Omega_{f}$ and $\Omega_{p}$, respectively, in the following numerical experiments.

### 4.1. Example 1

Assume that the computational domain $\Omega_{f}=\left(0,1\right)\times \left(1,2\right)$, $\Omega_{p}=\left(0,1\right)\times \left(0,1\right)$ and the interface $\Gamma =\left(0,1\right)\times \left\{1\right\}$. The physical parameter n=ρ=g=ν=K=α=1. The Dirichlet BCs and the forcing terms are given by the following exact solutions:

(26) $$\begin{array}{ccc} u_{f}\left(x\right) & = & (x^{2}{\left(y-1\right)}^{2},-\frac{2}{3}x{\left(y-1\right)}^{3}-\sin\left(x\right)), \end{array}$$

(27) $$\begin{array}{ccc} p_{f}\left(x\right) & = & xy-0.25, \end{array}$$

(28) $$\begin{array}{ccc} \varphi (x) & = & \sin\left(x\right)\left(y^{2}-y\right)+x-0.25. \end{array}$$

The relative $L_{2}$ error (25) of PPINNs and HC-PPINNs with three hidden layers and different numbers of neurons in each hidden layer $N_{neuron}$ are displayed in Table 1. From Table 1, we can see that, for HC-PPINNs and PPINNs, the relative $L_{2}$ error gradually decreases when $N_{neuron}$ increases. The approximated solutions obtained from HC-PPINNs are more accurate than those from PPINNs. In particular, the accuracy of HC-PPINNs with 8 neurons is higher than that of PPINNs with 32 neurons.

**Figure 4:**
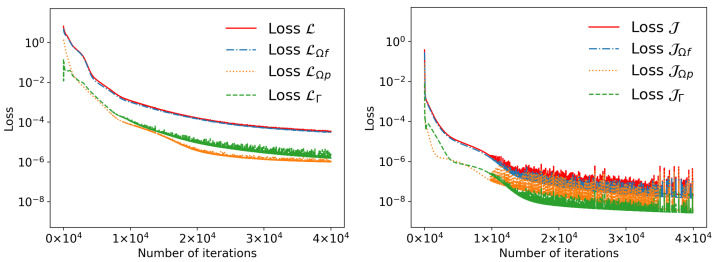
The loss history of Example 1, where both the neural networks have three hidden layers and 32 neurons in each hidden layer. (**Left**) Loss history of PPINNs. (**Right**) Loss history of HC-PPINNs.

Figure 4 shows the loss history of PPINNs and HC-PPINNs with three hidden layers and 32 neurons in each hidden layer. We can see that the training loss of HC-PPINNs is much lower than that of PPINNs.

**Figure 5:**
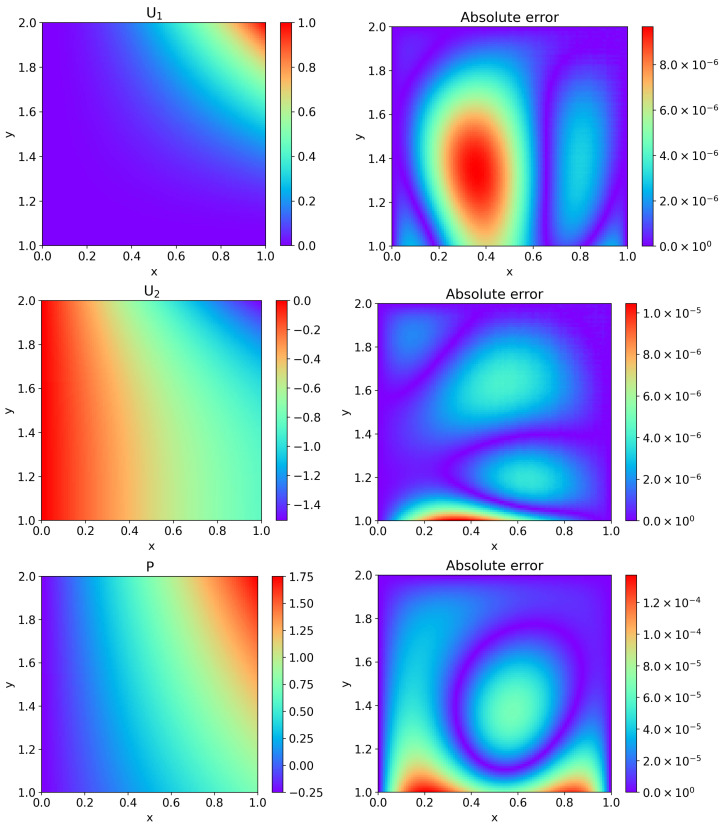

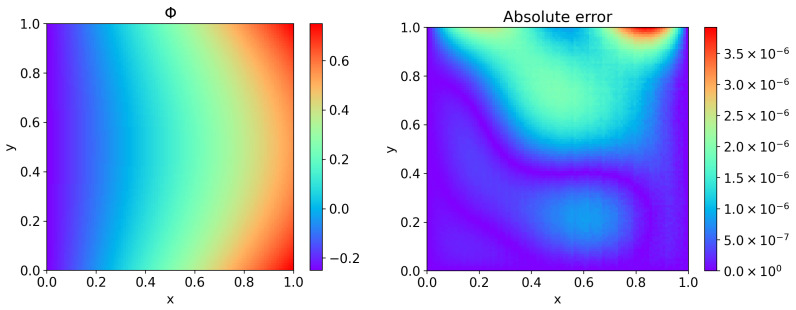
Comparison of the predicted values by HC-PPINNs and the exact solutions for Example 1, where the neural networks have three hidden layers and 32 neurons in each hidden layer. (**Left**) The left column is the predicted velocity $U_{1}$ in the *x* direction of fluid flow, the predicted velocity $U_{2}$ in the *y* direction of fluid flow, the predicted pressure P of fluid flow, and the predicted Φ of porous media flow, respectively. (**Right**) The right column is the corresponding absolute error between the predicted values and the exact solutions.

Figure 5 displays the predicted values by HC-PPINNs with three hidden layers and 32 neurons in each hidden layer, as well as the absolute error between the predicted values and the exact solutions. It can be observed that the predicted values approximate the exact solutions well.

### 4.2. Example 2

In this example, we consider the case with different values of the hydraulic conductivity *K*. The computational domain and the other physical parameters remain the same as in Example 1. The Dirichlet BCs and the forcing terms are chosen such that the exact solution of the coupled model is given by

(29) $$\begin{array}{ccc} u_{f}\left(x\right) & = & (y^{2}-2y+1,x^{2}-x), \end{array}$$

(30) $$\begin{array}{ccc} p_{f}\left(x\right) & = & 2\nu \left(x+y+1\right)+\frac{gn}{3K}, \end{array}$$

(31) $$\begin{array}{ccc} \varphi (x) & = & \frac{n}{K}\left(x\left(1-x\right)\left(y-1\right)+\frac{1}{3}y^{3}-y^{2}+y\right)+\frac{2\nu }{g}x. \end{array}$$

**Figure 6:**
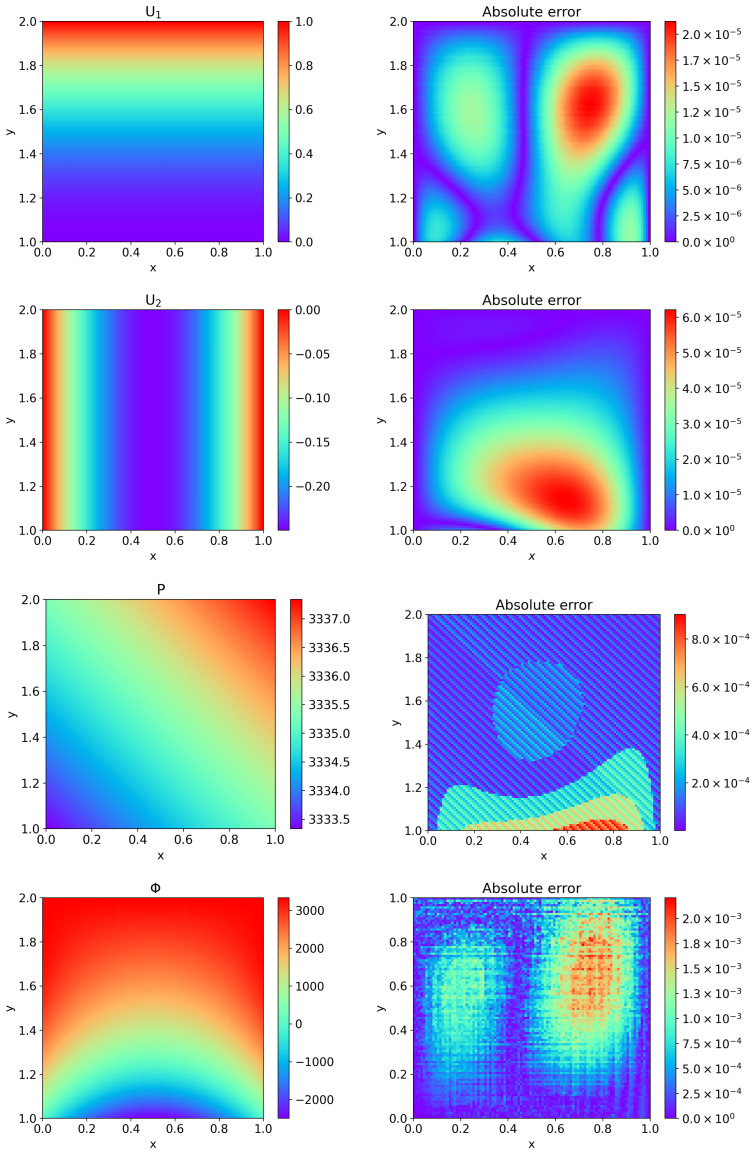
Comparison of the predicted values by HC-PPINNs and the exact solutions for Example 2 when K=0.0001, where the neural networks have three hidden layers and 16 neurons in each hidden layer. (**Left**) The left column is the predicted velocity $U_{1}$ in the *x* direction of fluid flow, the predicted velocity $U_{2}$ in the *y* direction of fluid flow, the predicted pressure P of fluid flow, and the predicted Φ of porous media flow, respectively. (**Right**) The right column is the corresponding absolute error between the predicted values and the exact solutions.

For HC-PPINNs, when K=0.001, the weight parameters ${\tilde{\lambda }}_{f}=1$, ${\tilde{\lambda }}_{p}=10$, ${\tilde{\lambda }}_{\Gamma }=1$. When K=0.0001, the weight parameters ${\tilde{\lambda }}_{f}=10$, ${\tilde{\lambda }}_{p}=100$, ${\tilde{\lambda }}_{\Gamma }=1$. For PPINNs, when K=0.01, the weight parameters $\lambda_{f}=1$, $\lambda_{p}=1$, $\lambda_{\Gamma }=10$. The relative $L_{2}$ error (25) of Example 2 with the varying hydraulic conductivity *K* is displayed in Table 2, where the neural networks have three hidden layers and 16 neurons in each hidden layer. From Table 2, we can see that HC-PPINNs can keep high accuracy when the hydraulic conductivity *K* becomes smaller, while PPINNs have lower accuracy even at K=0.01. We depict the predicted values of HC-PPINNs with three hidden layers and 16 neurons in each hidden layer, as well as the contrast between the exact solutions and the approximate solutions for K=0.0001 in Figure 6.

### 4.3. Example 3

**Figure 7:**
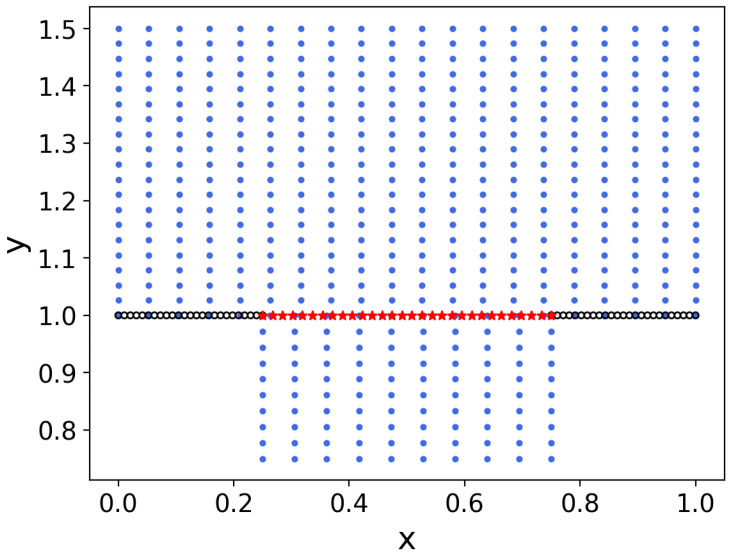
The sampling area and sampling points of Example 3. The black points ‘∘’ are boundary points, which will be treated in a “soft” manner, the red points ‘★’ are interface points, and the rest are residual points of equations.

Since PINNs is a mesh-free method for solving PDEs, in this example we consider an irregular computational domain to demonstrate that our method, HC-PPINNs, can still show good performance in this case. Let the computational domain $\Omega_{f}=\left(0,1\right)\times \left(1,1.5\right)$ and $\Omega_{p}=\left(0.25,0.75\right)\times \left(0.75,1\right)$ with the interface $\Gamma =\left(0.25,0.75\right)\times \left\{1\right\}$; see Figure 7. The physical parameters and the exact solutions remain the same as in Example 1.

Here, the boundary $\left\{(0,0.25),(0.75,1)\right\}\times \left\{1\right\}$ is on the same horizontal line as the interface $\Gamma =\left(0.25,0.75\right)\times \left\{1\right\}$; it will be difficult to construct $d_{f}\left(x\right)$ if this part of the boundary conditions are considered, so this part of the boundary conditions are incorporated into the loss in a “soft” manner.

We employ $N_{f}=400$, $N_{p}=100$, $N_{\Gamma }=30$, and $N_{bf}=50$. The sampling points are displayed in Figure 7. Then, we choose $d_{f}\left(x\right)=x\left(x-1\right)\left(y-1.5\right)$ and $d_{p}\left(x\right)=\left(x-0.25\right)\left(x-0.75\right)\left(y-0.75\right)$.

**Figure 8:**
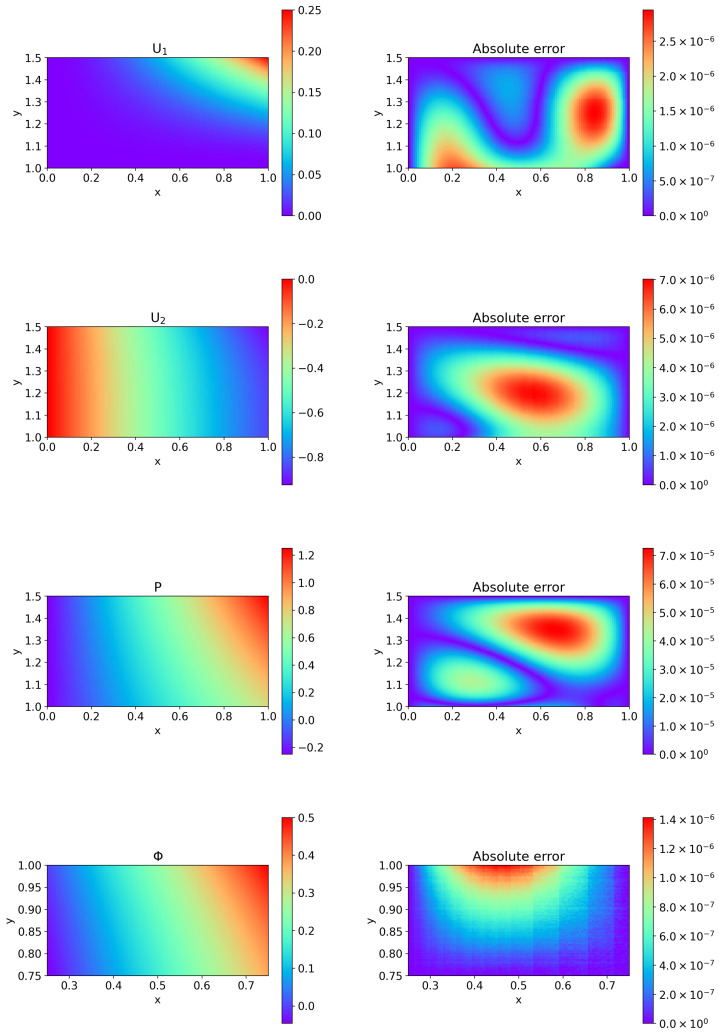
Comparison of the predicted values by HC-PPINNs and the exact solutions for Example 3, where the neural networks have three hidden layers and 32 neurons in each hidden layer. (**Left**) The left column is the predicted velocity $U_{1}$ in the *x* direction of fluid flow, the predicted velocity $U_{2}$ in the *y* direction of fluid flow, the predicted pressure P of fluid flow, and the predicted Φ of porous media flow, respectively. (**Right**) The right column is the corresponding absolute error between the predicted values and the exact solutions.

Table 3 shows the relative $L_{2}$ error (25) with three hidden layers and different numbers of neurons in each hidden layer, $N_{neuron}$. Figure 8 depicts the comparison of the predicted values of HC-PPINNs and the exact solutions, where the neural networks have three hidden layers and 32 neurons in each hidden layer. We can observe that the HC-PPINN still maintains high accuracy in the case of an irregular computational domain.

### 4.4. Example 4

**Figure 9:**
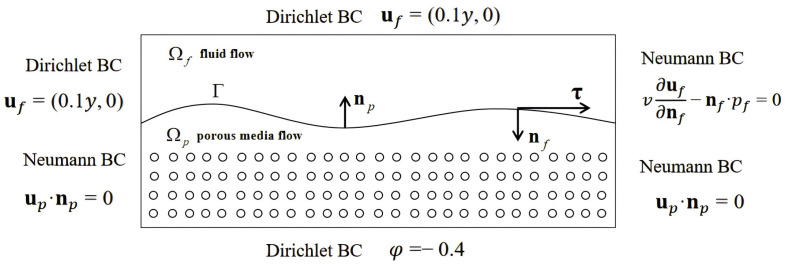
Boundary conditions of Stokes/Darcy problem.

In this example, we consider a more complex situation, the Stokes/Darcy model with mixed boundary conditions, to demonstrate the performance of HC-PPINNs. The computational domain and the parameters remain the same as in Example 1. The setting of the BCs is showed in Figure 9. We set $g_{f}$ = $g_{p}$ = 0.

Here, for HC-PPINNs, the Dirichlet BCs are incorporated into the loss in a “hard” manner, while the Neumann BCs are incorporated into the loss in a “soft” manner.

**Figure 10:**
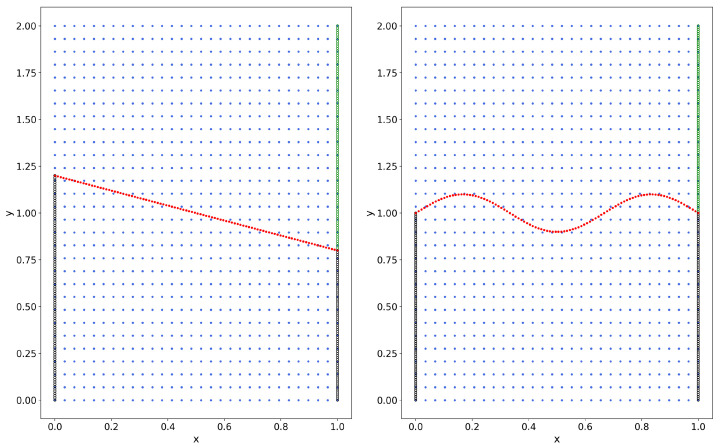
Sampling area and training points of Example 4, where the black and green points ‘∘’ are Neumann BCs points, the red points are interface points, and the rest are residual points of the equation. (**Left**) The straight-line interface. (**Right**) The curved interface.

**Figure 11:**
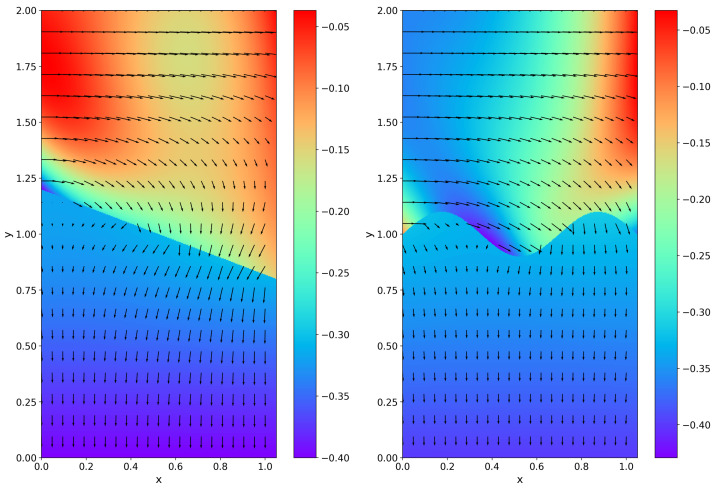
Simulation results of HC-PPINNs for Example 4, where the neural networks have three hidden layers and 16 neurons in each hidden layer. The color bar represent the approximated result of pressure and the vectors represent the velocity of the fluid. (**Left**) The straight-line interface. (**Right**) The curved interface.

We consider two different interface situations: one is the straight-line interface Γ: y+0.4x−1.2=0, and the other is the curved interface Γ:y=0.025sin(3πx). We employ $N_{f}=453$, $N_{p}=447$, $N_{bf}=100$, and $N_{bp}=175$ for the straight-line interface and $N_{f}=444$, $N_{p}=456$, $N_{bf}=100$, and $N_{bp}=200$ for the curved interface in the training. Figure 10 and Figure 11 show the training points and the simulation results, respectively. It can be seen that HC-PPINNs shows good performance for both straight-line and curved interfaces.

### 4.5. Example 5

In order to demonstrate that our method, HC-PPINNs, can greatly improve the accuracy of solving the Stokes/Darcy coupling problem, we consider the non-stationary mixed Stokes/Darcy model in this example. We consider the first test with nonhomogeneous boundary conditions as described in [46], and perform comparisons among HC-PPINNs, PPINNs, and Coupled Deep Neural Networks (CDNNs) in [46]. The computational domain is $\Omega_{f}=\left(0,1\right)\times \left(1,2\right)$, $\Omega_{p}=\left(0,1\right)\times \left(0,1\right)$ and the interface $\Gamma =\left(0,1\right)\times \left\{1\right\}$ with t∈(0,1]. All the physical parameters are set to 1. The boundary data and the forcing terms are chosen such that the exact solution of the coupled model is given by

(32) $$\begin{array}{ccc} u_{f}\left(x,t\right) & = & (\left[x^{2}{\left(y-1\right)}^{2}+y\right]\cos t,\left[-\frac{2}{3}x{\left(y-1\right)}^{3}+2-\pi \sin\left(\pi x\right)\right]\cos t), \end{array}$$

(33) $$\begin{array}{ccc} p_{f}\left(x,t\right) & = & \left[2-\pi \sin\left(\pi x\right)\right]\sin\left(\frac{\pi }{2}y\right)\cos t, \end{array}$$

(34) $$\begin{array}{ccc} \varphi \left(x,t\right) & = & [2-\pi \sin(\pi x)][1-y-\cos(\pi y)]\cos t. \end{array}$$

For HC-PPINNs, we choose $d_{f}\left(x,t\right)=x\left(x-1\right)\left(y-2\right)t$ and $d_{p}\left(x,t\right)=xy\left(x-1\right)t$. Thus, the initial conditions are also enforced in the loss function. We employ $N_{f}=N_{p}=512$, a learning rate of 0.001, and 20,000 iterations for both PPINNs and HC-PPINNs.

The relative $L_{2}$ errors (25) of HC-PPINNs, PPINNs, and CDNNs in [46] for Example 5 with different numbers of hidden layers and 16 neurons in each hidden layer are displayed in Table 4. We can see that, compared with CDNNs and PPINNs, HC-PPINNs are much more accurate.

## 5. Conclusions

In this paper, we aim to design HC-PPINNs to improve the accuracy of neural networks for solving Stokes/Darcy coupled problems. The method enforces the boundary conditions by changing the network architecture, and only the control equations as well as the interface equations need to be trained by incorporating them into the loss in a “soft” manner. Since it is not to be data driven, the training cost is greatly reduced. Through numerical experiments, the HC-PPINN has demonstrated that it can maintain good performance, not only in the regular region but also in the irregular region and the curved interface. And by comparing with PPINNs and CDNNs in [46], it is showed that HC-PPINNs greatly improve the network’s prediction accuracy. However, for the non-stationary mixed Stokes/Darcy coupling problem, our method does not have the extrapolation capability [52,53] to extend solutions to future time. This limitation arises because the treatment of temporal and spatial variables is consistent across all cases in HC-PPINNs, and further exploration is still needed to address this issue.

## Figures and Tables

**Table 1:** The relative $L_{2}$ error of PPINNs and HC-PPINNs for Example 1 with three hidden layers and different numbers of neurons in each hidden layer.

$N_{neuron}$	Algorithm	$u_{f}$	$p_{f}$	φ
8	PPINNs	4.97 × 10^−3^	6.52 × 10^−3^	2.71 × 10^−3^
	HC-PPINNs	4.15 × 10^−5^	7.01 × 10^−4^	1.02 × 10^−4^
16	PPINNs	1.84 × 10^−3^	2.63 × 10^−3^	1.51 × 10^−3^
	HC-PPINNs	2.64 × 10^−5^	2.12 × 10^−4^	1.60 × 10^−5^
32	PPINNs	1.71 × 10^−3^	3.17 × 10^−3^	1.65 × 10^−3^
	HC-PPINNs	6.68 × 10^−6^	9.23 × 10^−5^	5.62 × 10^−6^

**Table 2:** The relative $L_{2}$ error of Example 2 with the varying hydraulic conductivity *K*, where the neural networks have three hidden layers and 16 neurons in each hidden layer.

Algorithm	*K*	$u_{f}$	$p_{f}$	φ
HC-PPINNs	1	1.69 × 10^−5^	1.93 × 10^−5^	2.50 × 10^−6^
	0.1	3.48 × 10^−5^	1.75 × 10^−5^	2.60 × 10^−6^
	0.01	4.86 × 10^−5^	6.87 × 10^−6^	4.65 × 10^−6^
	0.001	6.56 × 10^−5^	6.39 × 10^−7^	1.12 × 10^−6^
	0.0001	6.17 × 10^−5^	6.06 × 10^−8^	4.59 × 10^−7^
PPINNs	0.01	4.66 × 10^−2^	1.22 × 10^−3^	4.73 × 10^−3^

**Table 3:** The relative $L_{2}$ error of HC-PPINNs for Example 3, where the neural networks have three hidden layers and a different number of neuron in each hidden layer.

$N_{neuron}$	$u_{f}$	$p_{f}$	φ
8	1.23 × 10^−5^	9.58 × 10^−5^	2.12 × 10^−6^
16	8.51 × 10^−6^	6.94 × 10^−5^	1.08 × 10^−6^
32	5.67 × 10^−6^	3.98 × 10^−5^	3.79 × 10^−6^

**Table 4:** The relative $L_{2}$ error of HC-PPINNs, PPINNs, and CDNNs in [46] for Example 5 with different numbers of hidden layers and 16 neurons in each hidden layer.

Number of Hidden Layers	Algorithm	$u_{f}$	$p_{f}$	φ
	HC-PPINNs	2.24 × 10^−5^	1.63 × 10^−4^	1.90 × 10^−5^
1	PPINNs	3.93 × 10^−2^	1.63 × 10^−1^	2.44 × 10^−1^
	CDNNs	1.25 × 10^−2^	1.87 × 10^−1^	8.02 × 10^−2^
	HC-PPINNs	2.34 × 10^−5^	1.68 × 10^−4^	1.64 × 10^−5^
2	PPINNs	1.71 × 10^−2^	6.32 × 10^−2^	7.40 × 10^−2^
	CDNNs	5.00 × 10^−4^	1.64 × 10^−2^	1.09 × 10^−3^
	HC-PPINNs	1.21 × 10^−5^	1.28 × 10^−4^	8.46 × 10^−6^
3	PPINNs	1.50 × 10^−2^	5.18 × 10^−2^	6.24 × 10^−2^
	CDNNs	1.15 × 10^−4^	3.14 × 10^−3^	2.28 × 10^−4^

## Data Availability

All data supporting the reported results are contained within the article itself. No external datasets or repositories were used, and no new data were generated that would require separate archiving.
